# Treatment Strategies that Enhance the Efficacy and Selectivity of Mitochondria-Targeted Anticancer Agents

**DOI:** 10.3390/ijms160817394

**Published:** 2015-07-29

**Authors:** Josephine S. Modica-Napolitano, Volkmar Weissig

**Affiliations:** 1 Department of Biology, Merrimack College, North Andover, MA 01845, USA; 2 Department of Pharmaceutical Sciences, Midwestern University, College of Pharmacy, Glendale, AZ 85308, USA; E-Mail: vweiss@midwestern.edu

**Keywords:** mitochondria, cancer, drug delivery systems, photodynamic therapy, combination therapy

## Abstract

Nearly a century has passed since Otto Warburg first observed high rates of aerobic glycolysis in a variety of tumor cell types and suggested that this phenomenon might be due to an impaired mitochondrial respiratory capacity in these cells. Subsequently, much has been written about the role of mitochondria in the initiation and/or progression of various forms of cancer, and the possibility of exploiting differences in mitochondrial structure and function between normal and malignant cells as targets for cancer chemotherapy. A number of mitochondria-targeted compounds have shown efficacy in selective cancer cell killing in pre-clinical and early clinical testing, including those that induce mitochondria permeability transition and apoptosis, metabolic inhibitors, and ROS regulators. To date, however, none has exhibited the standards for high selectivity and efficacy and low toxicity necessary to progress beyond phase III clinical trials and be used as a viable, single modality treatment option for human cancers. This review explores alternative treatment strategies that have been shown to enhance the efficacy and selectivity of mitochondria-targeted anticancer agents *in vitro* and *in vivo*, and may yet fulfill the clinical promise of exploiting the mitochondrion as a target for cancer chemotherapy.

## 1. Introduction

Despite enormous investments in the areas of basic research and medical science during the past few decades, cancer remains a leading health threat worldwide. Today in the United States alone, it is estimated that one in four adult men and one in five adult women are at risk of dying from cancer [1]. A resurgence of interest in the study of mitochondria has led to the discovery of several notable differences in the structure and function of this organelle between normal and cancer cells, and various attempts have been made to exploit these differences as novel and site specific targets for chemotherapy. Although a number of mitochondria-targeted compounds have shown some efficacy in selective cancer cell killing in pre-clinical and early clinical testing, the success of mitochondria-targeted therapeutic agents as a single modality treatment option for human cancers has been quite limited. This article presents an overview of mitochondria structure and function, especially as it relates to those differences found between normal and cancer cells, and highlights the progress made in exploiting this organelle as a target for chemotherapy. In addition, it summarizes three alternative treatment strategies that enhance the efficacy and selectivity of mitochondria-targeted anticancer agents *in vitro* and *in vivo* and offer the promise of therapeutic benefit. These include: mitochondria-targeted drug delivery systems; photodynamic therapy; and combination chemotherapy.

## 2. Mitochondria Structure and Function

In living cells, mitochondria are dynamic organelles comprising a network of long, filamentous structures that can be seen extending, contracting, fragmenting and fusing with one another as they move in three dimensions throughout the cytoplasm [2,3]. In electron micrographs of fixed tissue specimens, mitochondria appear as oval shaped particles similar in size to the bacterium *Escherichia coli* (1–2 microns long × 0.5–1.0 microns wide) and bound by two membranes. The outer membrane encloses the entire contents of the organelle. The inner membrane, which folds inward to form cristae, encloses the inner space, or matrix. Interestingly, the surface area of the inner mitochondrial membrane correlates with the degree of metabolic activity of the cell, and can vary considerably from cell type to cell type, or within a given cell depending upon its functional state. Mitochondria contain the enzymes and cofactors involved in a number of important metabolic reactions and pathways, including the tricarboxylic acid (TCA) cycle, oxidative phosphorylation, fatty acid degradation, the urea cycle, and gluconeogenesis. In mammalian cells, the matrix also typically contains up to 10,000 copies of a 16.6 kb closed circular double helical molecule of mitochondrial DNA (mtDNA), which is compacted *in vivo* to form a nucleoprotein complex, or nucleoid [4]. Although representing less than 1% of the total cellular DNA, mtDNA encodes two rRNAs, twenty-two tRNAs and thirteen highly hydrophobic polypeptide subunit components of four different respiratory enzyme Complexes (I, III, IV and V) that are localized to the inner mitochondrial membrane.

Mitochondria are considered the “powerhouse” of eukaryotic cells because of their central role in the process of aerobic metabolism. In carbohydrate metabolism, this begins when pyruvate, the end product of glycolysis, is transported from the cytosol into the mitochondrial matrix to undergo oxidative decarboxylation via the pyruvate dehydrogenase complex. In lipid metabolism, this begins when fatty acids are transported into the mitochondrial matrix to undergo sequential rounds of oxidative decarboxylation via the β-oxidation pathway. In either case, the resultant metabolic product is acetyl coA, which is further oxidized in the mitochondrial matrix via the TCA cycle. The net metabolic yield of the TCA cycle includes two molecules of CO_2_, one molecule of GTP (the energetic equivalent of ATP), three molecules of reduced nicotinamide adenine dinucleotide (NADH), and one molecule of reduced flavin adenine dinucleotide (FADH_2_). NADH and FADH_2_ go on to serve as respiratory substrates for oxidative phosphorylation, which couples the oxidation of these high-energy electron donors to the synthesis of ATP. In this process, electrons are transferred from NADH and FADH_2_ to oxygen via four multi-subunit electron transfer complexes located on the inner mitochondrial membrane. Complexes I, III and IV of the mitochondrial electron transfer chain assemble into functional supramolecular complexes, called respirasomes [5]. These three respiratory complexes also serve as proton pumps at which the energy derived from the transfer of electrons down the electron transport chain (ETC) is coupled to the translocation of protons from the matrix space outward to the space between the inner and outer mitochondrial membranes (*i.e.*, inter-membrane space). Under normal physiological conditions, the inner mitochondrial membrane is relatively impermeable to the backflow of protons and an electrochemical gradient is established across the membrane. The energy stored in this proton gradient, the proton-motive force, is then used to drive the synthesis of ATP from ADP and P_i_ via the inner membrane bound enzyme, mitochondrial ATP sythetase (Complex V). Oxidative phosphorylation supplies the vast majority of ATP produced by a cell under aerobic conditions.

Mitochondria are the main intracellular source of reactive oxygen species (ROS) in most tissues. It has been estimated that under physiological conditions, 1%–2% of the molecular oxygen consumed is converted to ROS molecules as a byproduct of oxidative phosphorylation [6]. ROS production can occur when a small fraction of reducing equivalents from Complex I or Complex III of the mitochondrial electron transport chain “leak” electrons directly to molecular oxygen, generating the superoxide anion O_2_^−^. Mitochondrial superoxide dismutase converts O_2_^−^ to H_2_O_2_, which can then acquire an additional electron from a reduced transition metal to generate the highly reactive hydroxyl radical ˙OH. There is increasing evidence that Complex II can also be a major regulator of mitochondrial ROS production under physiological and pathophysiological circumstances [7,8]. ROS play an important role as signaling molecules that mediate changes in cell proliferation, differentiation, and gene transcription [9,10]. Uncontrolled ROS activity, or oxidative stress, can damage intracellular protein and lipid components, and affect the integrity of biological membranes. High levels of ROS can also damage both nuclear and mtDNA. The mitochondrial genome is especially susceptible to ROS damage due to its proximity to the site of ROS production (*i.e.*, the ETC), as well as the fact that it has no introns or protective histones and a limited capacity for DNA repair. Thus, oxidative stress can impair mitochondrial function directly at the level of mitochondrial enzyme complexes, or as a consequence of its genotoxicity to mtDNA. Severe or prolonged oxidative stress can lead to irreversible oxidative damage and cell death [11].

Mitochondria also play a key role in mediating intrinsic apoptosis, an energy dependent cell death pathway regulated by numerous positive and negative signaling factors that exist in dynamic equilibrium [12]. Distally, intrinsic apoptosis can be induced by a variety of physiological or pathological cell stressors, such as toxins, viral infections, hypoxia, hyperthermia, free radicals, and DNA damage. Proximately, the intrinsic pathway is induced by the loss of anti-apoptotic proteins, (e.g., Bcl-2 and Bcl-x) or by activation of pro-apoptotic proteins (e.g., Bax and Bak). Intrinsic apoptosis involves mitochondrial outer membrane permeabilization (MOMP), the critical, irreversible step in the pathway that commits the cell to ultimate destruction. MOMP is followed by the release of cytochrome c and other apoptogenic proteins from the mitochondrial inter-membrane space. Once released into the cytosol, these proteins activate a caspase cascade, which leads to the proteolytic cleavage of intracellular proteins, DNA degradation, formation of apoptotic bodies, and other morphological changes that are considered hallmarks of apoptotic cell death. Both the intrinsic apoptotic pathway and the extrinsic apoptotic pathway, which involves cell membrane receptor-mediated interactions, play significant roles in normal development, tissue remodeling, aging, wound healing, immune response, and maintaining homeostasis in the adult human body.

## 3. Some Notable Differences between Mitochondria of Cancer Cells and Normal Cells

Nearly a century has passed since Otto Warburg first observed high rates of aerobic glycolysis in a variety of tumor cell types and suggested that this phenomenon might be due to an impaired respiratory capacity in these cells [13]. Warburg’s observations prompted many scientists to focus their investigative efforts on the mitochondria of cancer cells in an attempt to understand the underlying basis for the “Warburg Effect”, *i.e.*, enhanced glucose uptake, high rate of glycolysis in the presence of sufficient oxygen, and an increase in lactic acid as a byproduct of the glycolytic pathway. It is now known that at least some cancer cells possess a normal capacity for oxidative phosphorylation and can, under certain conditions, generate a majority of their ATP from this process [14,15,16,17,18,19,20,21]. In addition, recent evidence suggests that the enhanced glucose uptake and metabolic shift toward aerobic glycolysis in cancer cells is more likely due to their greater need for glucose metabolites, which serve as precursors for the biosynthesis of nucleic acids, amino acids, and lipids in these rapidly dividing cell populations [22], rather than to any specific impairment in respiratory function. In the years since Warburg’s initial observations, however, a number of notable differences between the mitochondria of normal and transformed cells have been identified [23,24,25,26,27,28]. These include differences in the size, number and shape of the organelle, the rates of protein synthesis and organelle turnover, and the polypeptide and lipid profiles of the inner mitochondrial membrane. Metabolic aberrations specifically associated with mitochondrial bioenergetic function in cancer cells include differences with regard to preference for respiratory substrates, rates of electron and anion transport, calcium uptake and retention, and decreased activities of certain enzymes integral to the process of oxidative phosphorylation, such as cytochrome c oxidase [29,30], adenine nucleotide translocase [31,32,33], and mitochondrial ATPase [34]. The mitochondrial membrane potential has also been shown to be significantly higher in carcinoma cells than in normal epithelial cells [35,36,37].

Alterations in mitochondrial genome sequence have also been linked to a variety of cancers [38,39,40]. Some are germ-line mutations. Among these, a human polymorphic variant in the NADH dehydrogenase 3 (*ND3*) gene at nt 10,398 (nt G10398A) that alters the structure of Complex I in the mitochondrial ETC was associated with an increased risk for invasive breast cancer in African–American women [38,41], the A12308G mutation in tRNA^Leu(CUN)^ was associated with increased risk of both renal and prostate cancers [42], and a variant in a non-coding region of mtDNA (16189T>C) was associated with increased susceptibility to endometrial cancer [43]. Somatic mutations in the mitochondrial genome are more common and have been observed in a wide variety of cancers, including ovarian, uterine, liver, lung, colon, gastric, brain, bladder, prostate, and breast cancer, melanoma and leukemia [26]. The displacement loop (or D-loop) region, a triple stranded non-coding sequence of mtDNA (np 16024-516) that houses cis regulatory elements required for replication and transcription of the molecule, has been shown to be a mutational “hot spot” in human cancer. However, mutations in genes encoding the polypeptide subunits of enzymes involved in oxidative phosphorylation also occur and can be of functional significance. Some of these are thought to be adaptive mutations that confer a selective advantage under the harsh growth conditions of the tumor microenvironment [40]. Others have been shown to be involved directly in tumor initiation and/or progression. For example, introduction of the pathogenic mtDNA *ATP6* T8993G mutation into the PC3 prostate cancer cell line through cybrid transfer produced tumors in nude mice that were 7-fold greater in size than those produced by wild-type cybrids [39]. Additionally, mutations in the mtDNA gene encoding NADH dehydrogenase subunit 6 (*ND6*) produced a deficiency in respiratory Complex I activity that was associated with an enhanced metastatic potential of tumor cells [44].

In general, tumor cells also exhibit higher levels of ROS than normal cells [9], and oxidative stress has been suggested to underlie the development and/or maintenance of the malignant phenotype. As noted previously, oxidative stress can cause somatic mutations in mtDNA. Evidence suggests that the converse is also true, *i.e.*, certain mutations in mtDNA, especially those in genes encoding ETC enzyme subunits, can cause ROS overproduction. Oncogene activation is also known to enhance the production of mitochondrial ROS, which has been implicated as a mechanism for K-RAS and MYC-mediated cell transformation [45,46]. In tumor cells, oxidative stress activates signaling pathways that promote cell growth and metastasis. One such pathway involves hypoxia-inducible factor (HIF), which regulates the transcription of a large number of genes that facilitate cell survival at low oxygen pressures [47]. Under the hypoxic conditions of tumor cell growth, mitochondria act as O_2_ sensors and further enhance ROS generation as an adaptive response [48]. ROS overproduction stabilizes the HIF-α subunit, facilitating its dimerization with the HIF-β subunit. This activates a number of different genes, including those mediating a metabolic shift toward glycolysis, angiogenesis, and metastasis. ROS have also been shown to activate MAP kinase and phosphoinositide 3-kinase pathways, which are important for cell proliferation and survival [9], and to up-regulate the expression of matrix metalloproteinases (MMPs) and Snail proteins, which are involved in epithelial-to-mesenchymal transition and metastasis, respectively [49].

Inhibition of the intrinsic apoptotic pathway is also observed in a number of hematopoietic malignancies and solid tumors, and has been implicated in cancer initiation, progression and metastasis [50,51]. This is thought to occur as a result of dysregulation of mitochondrial outer membrane proteins of the Bcl-2 family, and may involve overexpression or enhanced function of anti-apoptotic proteins, under-expression or loss of function of pro-apoptotic proteins, or a combination of both. For example, malignant chronic lymphocytic leukemia (CLL) cells express high levels of anti-apoptotic Bcl-2 and low levels of pro-apoptotic proteins such as Bax [52]. Interestingly, the progression of CLL is thought to be due to reduced apoptosis rather than increased proliferation *in vivo* [53]. Overexpression of Bcl-2 has also been shown to inhibit apoptosis in prostate [54], lung, colorectal and gastric cancers [55,56], neuroblastoma, glioblastoma, and breast carcinoma cells [57]. An imbalance in the expression of the anti- and pro-apoptotic Bcl-2 family of proteins is thought to stabilize the outer mitochondrial membrane, prevent MOMP and the release of cytochrome c, and ultimately, inhibit programmed cell death. This failure of normal cell turnover contributes to cell accumulation, transformation, and survival under extreme conditions, such as the hypoxic or acidic environments common in tumors. Interestingly, the inhibition of apoptosis that results from dysregulation of Bcl-2 protein expression has also been shown to underlie the development of drug resistance in cancer cells. For example, the overexpression Bcl-XL protects murine pro-lymphocytic cells from a wide variety of apoptotic stimuli and confers a multidrug resistance phenotype [58], and drug-induced apoptosis in B-CLL cells cultured *in vitro* is inversely related to Bcl-2/Bax ratios [52].

## 4. Mitochondria-Targeted Drugs that Show Selective Cancer Cell Killing

During the past few decades, scientists have been exploring the possibility that certain structural and functional differences that exist between the mitochondria of normal and transformed cells might serve as targets for selective cell killing by novel and site-specific anticancer agents. Recently, the term “mitocan” (an acronym for mitochondria and cancer) has been proposed to classify mitochondria-targeted anticancer agents, especially those that induce mitochondrial destabilization [59]. A number of these compounds have shown efficacy in selective cancer cell killing in pre-clinical and early clinical testing (see Table 1 for a representative sampling).

**Table 1:** Representative mitochondria-targeted compounds that exhibit selective cancer cell killing.

Class	Compound	Mode of Action	Demonstrated Efficacy	References
**OxPhos Inhibitors**	Rhodamine 123	ATP Synthase inhibitor	Preclinical (*in vitro*, *in vivo*)	[60,61,62]
	Dequalinium Chloride	Complex I inhibitor	Preclinical (*in vitro*, *in vivo*)	[63,64]
	AA-1	ATP Synthase inhibitor	Preclinical (*in vitro*, *in vivo*)	[65]
	MKT-077	General inhibition of ETC enzymes	Preclinical (*in vitro*, *in vivo*)	[66,67,68,69]
			Clinical, Phase I	
	Metformin	Complex I inhibitor	Preclinical (*in vitro*, *in vivo*)	[70,71,72,73,74,75,76,77,78,79,80,81,82,83,84,85,86,87,88,89]
			Clinical, Phase I	
**ROS Regulators**	Elesclomol	Enhanced ROS production	Preclinical (*in vitro*, *in vivo*)	[90,91,92]
			Clinical, Phase I	
	Bezielle	Enhanced ROS production	Preclinical (*in vitro*, *in vivo*)	[93,94,95,96,97,98,99]
			Clinical, Phase I	
**Intrinsic Apoptosis Inducers**	ABT-737	BH3 mimetic	Preclinical (*in vitro*, *in vivo*)	[100,101,102]
	ABT-263 (Navitoclax)	BH3 mimetic	Preclinical (*in vitro*, *in vivo*)	[103,104,105]
			Clinical, Phase I/II	
	Gossypol	BH3 mimetic	Preclinical (*in vitro*, *in vivo*)	[106,107]
	GX15-070 (Obatoclax)	BH3 mimetic	Preclinical (*in vitro*, *in vivo*)	[108,109]
	HA14-1	BH3 mimetic	Preclinical (*in vitro*, *in vivo*)	[110,111]

Among the earliest known mitochondria-targeted anticancer agents are the delocalized lipophilic cations (DLCs). Due to their lipophilicity and positive charge, these compounds selectively accumulate in the mitochondria of carcinoma cells in response to a higher, negative inside membrane potential (e.g., approximately 160 mV in carcinoma *vs.* 100 mV in control epithelial cells) [36,37]. Several DLCs have exhibited efficacy in carcinoma cell killing *in vitro* and *in vivo* [60,61,62,63,64,65,66,67,68,69,112,113], including the class prototype Rhodamine 123 (Rh123), dequalinium chloride (DECA), and the thiopyrylium AA-1. Although all DLCs are taken up into mitochondria by a common mechanism and display dose dependent mitochondrial toxicity, their specific mechanism of action can be quite varied. For example, Rh123 and AA-1 inhibit mitochondrial ATP synthesis at the level of F0F1-ATPase activity [62,65,113], while DECA and certain DLC thiacarbocyanines interfere with NADH-ubiquinone reductase (ETC Complex I) activity [64,112]. Another DLC, the water-soluble rhodacyanine dye analogue MKT-077, was shown to cause a more generalized deleterious effect on respiratory function through membrane perturbation and consequent inhibition of membrane-bound enzymes [67]. MKT-077 was the first DLC with a favorable pharmacological and toxicological profile and showed great promise as a selective anticancer agent in preclinical studies [66]. Phase I trials were undertaken to evaluate the safety and pharmacokinetics of MKT-077, but were halted due to recurrent but reversible renal toxicity in about half of the patients treated [68]. It was determined, however, that it is feasible to target mitochondria with rhodacyanine analogues if drugs with higher therapeutic indices could be developed [69].

More recently, evidence suggests that the widely prescribed anti-diabetic biguanide derivative, metformin, may also be effective in the prevention and treatment of human cancer via inhibition of mitochondrial respiratory function. Retrospective analyses show an association between the use of metformin and diminished cancer risk, progression and mortality in diabetic patients [70,71,72,73,74]. *In vitro* laboratory studies demonstrate that metformin has a direct and selective inhibitory effect on breast, colon, ovary, pancreas, lung, and prostate cancer cell lines [75,76,77,78,79]. In addition, at doses that had no effect on the viability of non-cancer stem cells, metformin inhibited transformation and selectively killed cancer stem cells resistant to chemotherapeutic agents [80]. *In vivo*, metformin inhibits the growth of spontaneous and carcinogen-induced tumors, and impacts tumor growth in mouse xenograft and syngeneic models [81,82,83,84,85]. Furthermore, prospective studies investigating the therapeutic efficacy of metformin use in non-diabetic cancer patients suggest its promise for the chemoprevention of colorectal cancer and treatment of early breast cancer [86,87,88]. It has been postulated that the therapeutic effects of metformin may be associated with both direct (insulin-independent) and indirect (insulin-dependent) actions of the drug [74]. However, results of a recent study showed that the direct inhibition of cancer cell mitochondrial Complex I by metformin was required to decrease cell proliferation *in vitro* and tumorigenesis *in vivo* [89]. Interestingly, it has been shown that cancer cell lines harboring mutations in mtDNA encoded Complex I subunits or having impaired glucose utilization exhibit enhanced biguanide sensitivity when grown under the low glucose conditions seen in the tumor microenvironment [114]. Metformin is a very safe and well-tolerated drug that is now prescribed to almost 120 million people in the world for the treatment of type II diabetes. Clinical trials using metformin alone and in combination with conventional anticancer agents in non-diabetic patients are ongoing and should clarify its potential use in cancer therapy.

Mitochondria-targeted ROS regulators have also shown efficacy as anticancer agents. Although the generally higher endogenous levels of ROS in tumor *versus* normal cells contribute to the development and/or maintenance of the malignant phenotype, they also render cancer cells more vulnerable to irreversible oxidative damage and consequent cell death. Therefore, pro-oxidant pharmacological agents that either enhance ROS production or inhibit ROS scavenging activity have the potential to increase ROS level beyond the threshold of lethality in cancer cells while leaving normal cells viable [115]. One such compound that targets mitochondria is elesclomol (STA-4783), an investigational, first-in-class small molecule that has been shown to enhance ROS production and induce a transcriptional gene profile characteristic of an oxidative stress response *in vitro*. Interestingly, the antioxidant *N*-acetylcysteine blocks elesclomol induced gene expression and apoptosis, indicating that ROS generation is the primary mechanism of cytotoxicity of the drug [115]. Comparative growth assays using the yeast model *S. cerevisiae* demonstrated that elesclomol interacts with the mitochondrial ETC to generate high levels of ROS and induce apoptosis [90]. In the same study, elesclomol was shown to interact similarly with the ETC in human melanoma cells. Elesclomol was granted fast-track designation by the FDA in 2006 for the treatment of metastatic melanoma. A randomized, double-blind, controlled SYMMETRY study evaluating the combination of paclitaxel and elesclomol in patients with advanced melanoma was stopped after all patients were enrolled because the addition of elesclomol to paclitaxel did not significantly improve progression free survival in unselected patients [91]. Studies are ongoing to determine the effect of elesclomol treatment alone and in combination with paclitaxel in patients with acute myeloid leukemia, and ovarian cancer [92].

Bezielle (BZL101), an aqueous extract from the herb *Scutellaria barbata*, is another ROS regulator that displays selective cytotoxicity against a variety of cancers *in vitro* and *in vivo* [93,94,95]. Early studies showed that in tumor cells, but not in non-transformed cells, Bezielle induces ROS production and causes severe DNA damage followed by hyperactivation of PARP-1, depletion of the cellular ATP and NAD, inhibition of glycolysis, and cell death [96]. It was later shown that treatment of tumor cells with Bezielle induces progressively higher levels of both mitochondrial superoxide and peroxide type ROS, and that Bezielle inhibits oxidative phosphorylation [97]. In addition, tumor cells lacking functional mitochondria did not generate mitochondrial superoxide and were protected from cell death in the presence of Bezielle, supporting the hypothesis that mitochondria are the primary target of the compound [97]. Bezielle has shown promising efficacy and excellent safety in the early phase clinical trials for advanced breast cancer [98,99].

Mitochondria-targeted compounds that induce outer membrane permeabilization and intrinsic apoptosis in cancer cells also show potential as anti-cancer agents. As previously discussed, BCL-2 family proteins, which share one or more of the four BCL-2 homology domains (BH1–BH4), regulate the intrinsic apoptotic pathway. Anti-apoptotic members of the family (such as BCL-2, BCL-X_L_, BCL-W and MCL-1), which are overexpressed in many cancers, function by sequestering the pro-apoptotic executioners of the MOMP (such as BAX and BAK). Inhibition of programmed cell death is antagonized by BH3-only proteins, a BCL-2 protein subfamily comprised of only the α-helical BH3 domain. These small proteins interact with anti-apoptotic molecules in their BH3-binding groove, causing the release and activation of BAX/BAK and inducing apoptosis [116]. Certain small molecules mimic the effect of BH3-only proteins. Among these BH3 mimetics, the synthetically derived ABT-737 has been shown to induce BAX/BAK-dependent apoptosis in a variety of cancer cell lines *in vitro*, and to display antitumor effects as a single agent *in vivo* [100,101,102]. Navitoclax (ABT-263), a potent, orally bioavailable analog of ABT-737 with similar biological activity, was shown to elicit complete tumor regression in small cell lung cancer (SCLC) and acute lymphoblastic leukemia xenograft models [103]. A phase I clinical study investigating the single-agent activity of navitoclax in the treatment of recurrent SCLC yielded encouraging preliminary safety and efficacy data [104]. However, in a subsequent phase II study navitoclax treatment induced only a low positive response and was limited by a dose-dependent and clinically significant thrombocytopenia [105]. Since both ABT-737 and navitoclax have been shown to potentiate the efficacy of standard cytotoxic agents against a variety of cancers [103,117,118,119,120,121], combinatorial regimens may ultimately prove a more promising therapeutic strategy for these compounds. Pre-clinical and clinical studies have shown that several other BH3 mimetics, such as the natural polyphenolic compound gossypol, and the synthetic compounds GX15-070 (obatoclax) and HA14-1 (ethyl 2-amino-6-bromo-4-(1-cyano-2-ethoxy-2-oxoethyl)-4*H*-chromene-3-carboxylate), also demonstrate anti-cancer activity, supporting the therapeutic potential of this class of mitochondria-targeted agents in the treatment of human cancer [106,107,108,109,110,111].

## 5. Alternative Treatment Strategies that Enhance the Efficacy and Selectivity of Mitochondria-Targeted Anticancer Agents

The fact that several mitochondria-targeted compounds have exhibited potent cancer cell killing in pre-clinical and early clinical studies is encouraging, and further research and testing of these compounds as viable, single modality treatment options for human cancers is warranted. However, the current limitations of this approach suggest the need also to explore the use of alternative treatment strategies in an effort to improve the efficacy and selectivity of these anticancer agents. Presented below (and summarized in Table 2) are three treatment strategies that have been shown *in vitro* and *in vivo* to enhance the selective cancer cell killing of several compounds known to have direct or indirect effects on mitochondrial function. It is proposed that by expanding the application of these strategies to include additional mitochondria-targeted compounds already known to exhibit significant preclinical and clinical anticancer activity as single agents (e.g., oxidative phosphorylation inhibitors, ROS regulators, and apoptosis inducers), the therapeutic efficacy of these compounds might also be improved.

**Table 2:** Treatment strategies that have been shown to enhance the efficacy and selectivity of anticancer agents.

Strategy	Carrier/Class	Anticancer Agent	References
**Mitochondria-Targeted Drug Delivery Systems**	TPP^+^-conjugated molecules	Vitamin E succinate	[122,123]
		Coenzyme Q	[124]
	DQAsomes	Paclitaxel	[125,126,127]
		Curcumin	[128]
		Resveratrol	[129]
	STPP^+^ liposomes	Paclitaxel	[130,131]
		Doxorubicin	[132]
	Mito-targeted nanontubes	Platinum (IV)	[133]
**Photodynamic Therapy**	Cationic photosensitizers	EDKC	[134]
		Rh123	[135]
		MKT-077	[136]
	Non-cationic photosensitizers	Pba	[137,138,139,140,141,142,143]
		BBr2	[144]
**Combination Chemotherapy**	Inhibitors of glycolysis and oxidative phosphorylation	2-DG plus metformin	[145,146]
	Inhibitors of two or more mitochondrial target sites	AZT plus MKT-077	[147]

### 5.1. Mitochondria-Targeted Drug Delivery Systems

Over the past several decades, attempts have been made to develop mitochondriotropic drug delivery systems for a variety of therapeutic purposes. One early strategy employed mitochondrial protein-import machinery to deliver macromolecules to mitochondria. For example, a mitochondrial signal sequence was used to direct green fluorescent protein to mitochondria to allow the visualization of mitochondria within living cells [148]. Another strategy employed conjugation with well-established mitochondriotropic cations, such as triphenylphosphonium (TPP^+^) to successfully target low-molecular weight molecules to mammalian mitochondria. These molecules rapidly permeate lipid bilayers and, in response to the plasma and mitochondrial membrane potentials (negative inside), accumulate several hundredfold inside the organelle. One study demonstrated that significant doses of the TPP-conjugated antioxidants coenzyme Q or vitamin E could be fed safely to mice over long periods, and achieve steady-state distributions within the heart, brain, liver, and muscle [149]. These results showed that mitochondria-targeted bioactive molecules can be administered orally, leading to their accumulation at potentially therapeutic concentrations in those tissues most affected by mitochondrial dysfunction. More recently, mitochondria-targeted, TPP-conjugated vitamin E succinate has been shown to act preferentially on cancer cells, suppressing mitochondrial function and mtDNA transcription and blocking proliferation at low concentrations [122], and inducing apoptosis at higher concentrations [123]. In another study, Mito-Q (coenzyme-Q conjugated to an alkyl triphenylphosphonium cation) and Mito-CP (a 5-membered nitroxide, CP, conjugated to a TPP cation) potently inhibited the proliferation of breast cancer cells (MCF-7 and MDA-MB-231) [124] and human colon cancer cells (HCT-116) [45], further demonstrating the anticancer potential of TPP-conjugated molecules.

A quantitative structure activity relationship (QSAR) model was developed to facilitate guided synthesis and selection of optimal mitochondriotropic structures [150]. In theory, any compound that acts on mitochondria can be chemically modified to become mitochondriotropic. However, there are limitations to this strategy. First, not all potentially therapeutic compounds with molecular targets at or inside mammalian mitochondria find their way to mitochondria once inside a cell. This is because the intracellular distribution of a low-molecular weight compound is strongly affected not only by its own physico-chemical properties, but also by the cytoskeletal network, dissolved macromolecules, and dispersed organelles. Furthermore, any chemical modification that renders a compound mitochondriotropic may adversely affect its inherent pharmacological activity. In contrast, pharmaceutical nanocarriers offer an alternative approach to improve the intracellular disposition of potentially therapeutic compounds. The benefit of this strategy is that all chemistry can be carried out on the components of the nanocarrier, leaving the pharmacological profile of the compound unaltered [151]. Furthermore, nanocarrier delivery can overcome several limitations for the therapeutic use of free compounds, such as lack of water solubility, non-specific biodistribution and targeting, and low therapeutic indices.

The idea that nanocarriers could serve as effective mitochondria-targeted drug delivery systems arose in the late 1990s with the accidental discovery of the vesicle-forming capacity of dequalinium chloride, a cationic bolaamphiphile comprising two quinaldinium rings linked by ten methylene groups [152]. The compound was found to self-assemble into liposome-like vesicles, called DQAsomes (DeQAlinium-based lipoSOMES), and to have a strong affinity for mitochondria [153,154]. Follow-up studies confirmed the suitability of DQAsomes for the delivery of bioactive compounds to mitochondria, and DQAsomes are now considered the prototype for all vesicular mitochondria-specific nanocarriers [155]. *In vitro* and *in vivo* studies have shown that DQAsomal preparations of the anticancer agent paclitaxel increase the solubility of the drug by a factor of 3000, and enhance its efficiency in triggering apoptosis by direct action on mitochondria [125,126,127]. More recently, DQAsomes have been used for the pulmonary delivery of curcumin [128], a potent antioxidant with anti-inflammatory and potential anticancer properties. Due to its water-insolubility, however, curcumin’s bioavailability following oral administration is extremely low. Curcumin encapsulated into DQAsomes displays enhanced antioxidant activity in comparison to the free compound.

Interestingly, a mitochondria-targeting drug delivery system in which dequalinium chloride has been covalently linked to the hydrophilic distal end of polyethylene glycol-distearoylphosphatidylethanolamine (DQA-PEG(2000)-DSPE) has also been prepared [129]. These nanocarriers were used to deliver resveratrol to mitochondria in human lung adenocarcinoma A549 cells, resistant A549/cDDP cells, A549 and A549/cDDP tumor spheroids as well as the xenografted resistant A549/cDDP cancers in nude mice. Results demonstrated that the mitochondrial targeting of resveratrol induced apoptosis in both non-resistant and resistant cancer cells by dissipating the mitochondria membrane potential, releasing cytochrome c and increasing the activities of caspase 9 and 3 [129]. DQAsomes have also been used to deliver an artificial mini-mitochondrial genome construct encoding Green Fluorescence Protein (GFP) to the mitochondrial compartment of a mouse macrophage cell line resulting in the expression of GFP mRNA and protein [156]. Though the transfection efficiency for GFP was very low this work constitutes the very first reported successful transgene expression inside mitochondria within living mammalian cells.

Conventional liposomes are another type of pharmaceutical nanocarrier that can also be rendered mitochondria-specific via the surface attachment of known mitochondriotropic residues, such as the cation TPP [157,158,159,160]. Preparation of liposomes in the presence of hydrophilic molecules, which have been artificially hydrophobized via linkage to fatty acid or phospholipid derivatives, results in the covalent “anchoring” of the hydrophilic moiety to the liposomal surface [161,162]. In 2005, TPP cations were conjugated to stearyl residues (yielding stearyl-TPP, or STPP), and STPP-bearing liposomes were first shown to exhibit *in vitro* mitochondriotropism [157]. The same group later demonstrated that surface modification of nanocarriers with mitochondriotropic TPP cations facilitates the efficient subcellular delivery of a model compound, ceramide, to mitochondria of mammalian cells and improves its cytotoxic and pro-apoptotic activities *in vitro* and *in vivo* [158]. More recently, STPP liposomes have been used as nanocarriers to enhance the efficacy of mitochondria-targeted anticancer agents. For example, paclitaxel loaded STPP liposomes were shown to co-localize with mitochondria and to significantly increase cytotoxicity by paclitaxel in a drug resistant ovarian carcinoma cell line [130]. The improvement in cytotoxicity was found to result from the increased accumulation of paclitaxel in mitochondria, as well as from the specific toxicity of STPP towards the resistant cell line. Mechanistic studies revealed that the cytotoxicity of STPP was associated with a decrease in mitochondrial membrane potential and other hallmarks related to caspase-independent cell death. Interestingly, mitochondriotropic STPP liposomes can be made to exhibit even greater cancer cell specificity with the addition of another ligand, folic acid. Cancer cell-specific targeting via surface modification with these dual ligands has been shown to enhance the cellular and mitochondrial delivery of doxorubicin in KB cells, and produce a synergistic effect on ROS production and cytotoxicity in this tumor cell line [132].

The preparation of TPP-surface modified liposomes utilizing an alternative hydrophobic anchor for TPP cations has also been described. For example, a d-alpha-tocopheryl polyethylene glycol 1000 succinate-triphenylphosphine conjugate (TPGS1000-TPP) was synthesized as the mitochondrial targeting molecule and incorporated into the membranes of paclitaxel-loaded liposomes [131]. The paclitaxel loaded TPGS1000-TPP conjugated liposomes were shown to selectively accumulate in the mitochondria. This targeted delivery of paclitaxel caused the release of cytochrome c, initiated a cascade of caspase 9 and 3 reactions, and enhanced apoptosis by activating pro-apoptotic pathways and inhibiting anti-apoptotic pathways. In comparison with taxol and regular paclitaxel liposomes, the mitochondria targeted paclitaxel liposomes exhibited the strongest anticancer efficacy against drug resistant lung cancer cells *in vitro* and in a nude mouse xenograft model *in vivo*, suggesting a potential therapeutic treatment for drug-resistant lung cancer.

A number of other TPP^+^ modified nanocarriers have shown promise as effective mitochondrial specific drug delivery systems. One novel mitochondriotropic nanocarrier based on an oligolysine scaffold with the addition of two triphenylphosphonium cations per oligomer, and another based on a 5 poly(amidoamine) dendrimer conjugated with TPP^+^, were shown to be efficiently taken up by cells and display a high degree of mitochondrial specificity [163,164]. A TPP-conjugated, mitochondria-targeted nano delivery system for coenzyme Q10 (CoQ10) has also been shown to reach mitochondria and to deliver CoQ10 in adequate quantities [165]. The multifunctional nanocarrier is composed of poly(ethylene glycol), polycaprolactone and triphenylphosphonium bromide and was synthesized using a combination of click chemistry with ring-opening polymerization followed by self-assembly into nanosized micelles. A potential disadvantage of this system, however, is the localization of the mitochondrial targeting moiety, which is seated between the two polymers, *i.e.*, between the poly(ethylene glycol) and polycaprolactone units. In a different approach, TPP^+^ was linked to the PEG side of a PLGA-PEG-based block copolymer, thereby enhancing the availability of the targeting moiety for any potential interaction with mitochondrial membranes [166]. In a follow-up study, Zinc phtalocyanine (ZnPc) was encapsulated inside PLGA-b-PEG-TPP polymer nanoparticles. By targeting ZnPc to the mitochondria, singlet oxygen was locally produced inside the mitochondria to effectively initiate apoptosis [167]. Interestingly, TPP-conjugated poly(ethylene imine) hyperbranched polymer nanoassemblies were also shown to successfully deliver doxorubicin to the mitochondria of human prostate carcinomas cells and cause rapid and severe cytotoxicity within few hours of incubation, even at sub-micromolar incubation concentrations [168].

The mitochondrial cationic dye, rhodamine-110, has also been used for rendering carbon nanotubes (CNTs) mitochondriotropic. In one study, multi-walled carbon nanotubes (MWCNTs) were functionalized with either mitochondrial-targeting fluorescent rhodamine-110 (MWCNT-Rho) or non-targeting fluorescein (MWCNT-Fluo) as a control [133]. Results demonstrated that MWCNT-Rho co-localized well with mitochondria (*ca.* 80% co-localization) in contrast to MWCNT-Fluo, which showed poor association with mitochondria (*ca.* 21% co-localization). In addition, platinum (IV), a prodrug of cis-platin, displayed significantly enhanced cytotoxicity towards several cancer cell lines when incorporated into mitochondria-targeted carbon nanotubes in comparison to non-targeted formulations [133]. MWCNTs have also been functionalized with peptides having a mitochondria-targeted peptide sequence (MTS). The association of such MWCNT-MTS conjugates with mitochondria inside murine macrophages and HeLa cells has been confirmed by wide-field epifluorescence microscopy, confocal laser scanning microscopy and transmission electron microscopy (TEM). The localization of the MTS-MWCNT conjugates with mitochondria was further confirmed by analyzing the isolated organelles using TEM [169]. The use of nanoparticles for the delivery of small molecule anticancer agents has thus shown past success and holds much promise for further development and therapeutic application.

### 5.2. Photodynamic Therapy

Photodynamic therapy (PDT) involves the use of a photoreactive drug, or photosensitizer, that is selectively taken up or retained by target cells or tissues. Upon administration of light of a specific wavelength, the photosensitizer becomes activated from a ground state to an excited state. As the photosensitizer returns to the ground state, the energy is transferred to molecular oxygen, thus generating ROS and inducing cellular toxicity in the particular areas of tissue that have been exposed to light [170]. There has been considerable interest in PDT as a treatment modality for a variety of cancers [170,171]. Photofrin, which was first used in PDT in 1993 for the prophylactic treatment of bladder cancer, is the most common photosensitizer in clinical use today. However, a number of other photosensitizers have been approved for clinical use or have undergone clinical testing to treat cancers of the head and neck, brain, lung, pancreas, intraperitoneal cavity, breast, prostate and skin. The selectivity of a photosensitizer and its site of action within a cell contribute to the efficacy of PDT. Evidence suggests that subcellular localization is more important than photochemical reactivity in terms of overall cell killing, and that mitochondrial localization represents a highly desirable property for the development of highly specific and efficient photosensitizers for photodynamic therapy applications [172].

Cationic photosensitizers are particularly promising as potential PDT agents. Like other DLCs, these compounds are concentrated by cells and into mitochondria in response to negative-inside transmembrane potentials, and are thus selectively accumulated in the mitochondria of carcinoma cells. In combination with localized photoirradiation, the cationic photosensitizer can be converted to a reactive and highly toxic species, thus enhancing its selectivity for and toxicity to carcinoma cells, and providing a means of highly specific tumor cell killing without injury to normal cells. Several cationic photosensitizers have shown promise for use in PDT. For example, selective photoxicity of carcinomas *in vitro* and *in vivo* has been observed for a series of triarylmethane derivatives [173] and the kryptocyanine EDKC [134]. Both Rh123 and the chalcogenapyrylium dye 8b have been evaluated as photosensitizers for the photochemotherapy of malignant gliomas [135,174]. In another study, photoactivation of the selective anticancer agent MKT-077 was shown to enhance its mitochondrial toxicity [136]. As expected, the mechanisms of mitochondrial toxicity exhibited by these compounds are varied, and range from specific inhibition of mitochondrial enzymes to non-specific perturbation of mitochondrial function due to singlet oxygen production.

Non-cationic photosensitizers that target mitochondria have also shown promise for use in PDT. Pheophorbide a (Pba), is a chlorophyll breakdown product isolated from silkworm excreta and the Chinese medicinal herb, Scutellaria barbarta [137,175]. Because Pba absorbs light at longer wavelengths than the first-generation photosensitizer photofrin, tissue penetration is enhanced. Pba has been shown to accumulate in mitochondria and cause apoptosis in a variety of cancer cells, including leukemia, and uterine, breast, pancreatic, colon and hepatocellular carcinoma [137,138,139,140,141,142,143]. *In vivo* animal studies have supported the efficacy of Pba-PDT in preventing tumor cell growth. [139,143]. In addition, the tetra-aryl brominated porphyrin and the corresponding diaryl derivative are also promising sensitizers with good photodynamic properties that have the ability to accumulate in mitochondria and induce cell death in human melanoma and colorectal adenocarcinoma *in vitro* and *in vivo* [144]. These results have positive implications for the use of mitochondria-targeted PDT compounds in cancer therapy.

### 5.3. Combination Chemotherapy

As noted previously, the two major pathways for cellular ATP production are glycolysis and mitochondrial oxidative phosphorylation. The high rate of aerobic glycolysis in cancer cells makes them particularly vulnerable to chemotherapeutic agents that inhibit glycolytic enzymes. For example, 2-deoxy-d-glucose (2DG), 3-bromopyruvate (3-BrPA), and lonidamine, which inhibit the hexokinase (HK) catalyzed first step in glycolysis, each have demonstrated significant anticancer activity against a variety of cell types *in vitro* and *in vivo* [176,177,178,179,180,181]. Unfortunately, the therapeutic efficacy of these compounds as single agents appears to be quite limited. Perhaps this is due to the fact that many cancer cells have functionally competent mitochondria and can overcome inhibition of the glycolytic pathway by increasing mitochondrial ATP production.

Recent evidence suggests that combination chemotherapy, simultaneously aimed at both glycolytic and mitochondrial pathways for ATP production, can be a more effective chemotherapeutic approach for the selective cytotoxicity of cancer cells. In one study [145], the *in vitro* antitumor activity 2DG alone was found insufficient to promote tumor cell death in human breast cancer and osteosarcoma cell lines, reflecting its limited efficacy in clinical trials. However, the combination of 2DG and metformin led to significant cell death associated with a decrease in cellular ATP. Gene expression analysis and functional assays revealed that metformin compromised OXPHOS. Furthermore, forced energy restoration with methyl pyruvate reversed the cell death induced by 2DG and metformin, suggesting a critical role of energetic deprivation in the underlying mechanism of cell death. The combination of 2DG and metformin also inhibited tumor growth and metastasis in mouse xenograft tumor models [145]. In another study, the combination of 2DG and metformin was shown to inhibit both mitochondrial respiration and glycolysis in prostate cancer cells leading to a severe depletion in cellular ATP. This combination of drugs induced a 96% inhibition of cell viability in LNCaP prostate cancer cells, a cytotoxic effect that was much greater than that induced by treatment with either drug alone. In contrast, only a moderate effect by the combination of 2DG and metformin on cell viability was observed in normal prostate epithelial cells [146].

The selective tumor cell killing by mitochondria-targeted DLCs can also be enhanced by combination with anticancer agents having alternative mitochondria target sites. For example, 3-azido deoxythymidine (AZT) as a single agent was found to induce a dose-dependent inhibition of cell growth of several human carcinoma cells, yet cause no significant effect on the growth of control epithelial cells [147]. Combination treatment employing a constant concentration of a delocalized lipophilic cation (dequalinium chloride or MKT-077) plus varying concentrations of AZT enhanced the AZT-induced cytotoxicity of carcinoma cells up to four-fold. The drug combination of constant DLC and varying AZT had no significant effect on the growth of control cells. Furthermore, clonogenic assays demonstrated up to 20-fold enhancement of selective carcinoma cell killing by combination *vs.* single agent treatment, depending on the specific drug combination and concentrations used. It was hypothesized that the efficacy of the AZT/DLC drug combination in carcinoma cell killing may be based on a dual selectivity involving inhibition of mitochondrial energy metabolism and inhibition of DNA synthesis due to limited deoxythymidine monophosphate availability [147].

Although limited in scope and number, the results of these drug combination studies are encouraging. More importantly, they suggest that additional studies should be undertaken to assess the anticancer activity of novel combinations of metabolic inhibitors targeting both major pathways of ATP production, and of novel combinations of compounds that target different sites in mitochondria.

## 6. Summary and Concluding Remarks

A persistent challenge in cancer therapy is to find ways to improve the efficacy and selectivity of a therapeutic compound while minimizing its systemic toxicity and treatment-limiting side effects. The central role that mitochondria play in the life and death of a cell, together with the many differences found to exist between the mitochondria of normal and transformed cells, make them prime targets for anticancer agents. However, despite the fact that a number of mitochondria-targeted compounds have exhibited potent and selective cancer cell killing in preclinical and early clinical testing, currently none has achieved the standards for high selectivity and efficacy and low toxicity necessary to progress beyond phase III clinical trials and to be used as a viable, single modality treatment option for human cancers. The limitations of this approach suggest the need to explore the use of alternative treatment strategies to enhance the efficacy and selectivity of mitochondria-targeted anticancer agents. Mitochondria-targeted drug delivery systems, photodynamic therapy, and combination chemotherapy are three strategies that have been shown to enhance the efficacy and selectivity of certain mitochondria-targeted anticancer agents *in vitro* and *in vivo*. These strategies enhance the effects of potential therapeutic agents either by delivering them directly to the site of action (mitochondria-targeted drug delivery systems), or by increasing their potency once they have reached their target site (PDT, combination chemotherapy). It is proposed that by expanding the application of these strategies to include additional mitochondria-targeted compounds that have already demonstrated significant preclinical and clinical anticancer activity as single agents, including but not limited to those summarized in this review, the therapeutic efficacy of these compounds might also be improved. New and ongoing research in this area is warranted, and may yet fulfill the clinical promise of exploiting the mitochondrion as a target for cancer chemotherapy.
